# Biodegradation of plastics in soil and effects on nitrification activity. A laboratory approach

**DOI:** 10.3389/fmicb.2014.00710

**Published:** 2014-12-15

**Authors:** Giulia Bettas Ardisson, Maurizio Tosin, Marco Barbale, Francesco Degli-Innocenti

**Affiliations:** Ecology of Products and Environmental Communication, Novamont S.p.A., NovaraItaly

**Keywords:** bioplastics, biodegradation, biodegradability, soil ecotoxicity, soil nitrification, biodegradable materials, soil microbiology

## Abstract

The progressive application of new biodegradable plastics in agriculture calls for improved testing approaches to assure their environmental safety. Full biodegradation (≥90%) prevents accumulation in soil, which is the first tier of testing. The application of specific ecotoxicity tests is the second tier of testing needed to show safety for the soil ecosystem. Soil microbial nitrification is widely used as a bioindicator for evaluating the impact of chemicals on soil but it is not applied for evaluating the impact of biodegradable plastics. In this work the International Standard test for biodegradation of plastics in soil (ISO 17556, 2012) was applied both to measure biodegradation and to prepare soil samples needed for a subsequent nitrification test based on another International Standard (ISO 14238, 2012). The plastic mulch film tested in this work showed full biodegradability and no inhibition of the nitrification potential of the soil in comparison with the controls. The laboratory approach suggested in this Technology Report enables (i) to follow the course of biodegradation, (ii) a strict control of variables and environmental conditions, (iii) the application of very high concentrations of test material (to maximize the possible effects). This testing approach could be taken into consideration in improved testing schemes aimed at defining the biodegradability of plastics in soil.

## INTRODUCTION

Plastic materials are used in several applications in modern agriculture. For example, plastic mulch films made with polyethylene are successfully applied to control weeds and stabilize soil temperature and moisture. In order to maintain the advantages conferred by using plastic products without having the burden of final waste management, plastic mulch films have been developed that are designed for biodegradation in soil and are used in place of traditional non-biodegradable mulch films. The intentional release of biodegradable plastics in soil implies that two aspects are satisfied: full biodegradability and no ecotoxic effects on soil organisms. Testing schemes and acceptability criteria based on these principles have already been developed: the “OK Biodegradable soil” Certification by the Belgian Institute Vinçotte; the French standard NF U 52-001 (2005), the Italian standards UNI 11462 (2012), and UNI 11495 (2013).

The “OK Biodegradable soil” and the Italian standards set the biodegradation level to be reached in 2 years at 90%, whereas the AFNOR standard has a more articulated testing scheme still based on the achievement of a biodegradation level equal to or greater than 90%. The 90% biodegradation level is a very stringent requirement for showing plastics biodegradability. Aerobic biodegradation is the conversion of plastic material into carbon dioxide, water, and biomass (i.e., the new biological matter developed by the growing microorganisms) with the consumption of atmospheric oxygen:

(1) $$C_{plastic}+O_{2}\to {CO}_{2}+H_{2}O+C_{residue}+C_{biomass}$$

The test methods for measuring biodegradation are based on respirometry. Respirometric test methods can only assess one carbonic product of the reaction (CO_2_). No reliable methods to determine the biomass (C_biomass_) or the by-products (C_residue_) produced during biodegradation are available yet. Therefore, the biomass and potential by-products are excluded from the equation, preventing a 100% mass balance being achieved. As a result, a complete biodegradation of “C_plastics_” will rarely lead to a complete mineralization (i.e., to a carbon dioxide evolution corresponding to the maximum amount expected in the event of total conversion of the “C_plastics_” into CO_2_) because at least part of the original plastic material will be converted into biomass as a result of anabolic biochemical pathways. For these reasons, a mineralization value equal to or greater than 90% is considered by the scientific community to be proof of complete biodegradation, not only in the field of biodegradable plastics but also in other sectors such as detergency.

The “OK Biodegradable soil” and the Italian and French standards also include ecotoxicity requirements. Ecotoxicity tests must be carried out in soil samples where plastic material added at 1% concentration has been degraded for 3 months. Ecotoxicity test methods currently applied by the standards are: the determination of seed germination and growth of plants (UNI 10780, 1998; ISO 11269-2, 2012); acute toxicity on earthworms (FD X 31-251, 1994; ISO 11268-1, 2012); acute toxicity on aquatic organisms such as Daphnia (ISO 6341, 2012) or algae (NF T 90-375, 1998). Recently it has been suggested that microbial nitrification could also be included as a bioindicator to evaluate the impact of biodegradable polymers in soil (Chenon et al., 2012).

Nitrification is important for the health of the soil and the ecosystem because it completes the mineralization of organic nitrogen started with the ammonification process, a relevant process in the nitrogen cycle. Ammonium is converted into nitrates by two consecutive steps performed by two groups of microorganisms: the ammonia-oxidizing bacteria (AOB) and the ammonia-oxidizing archaea (AOA), that perform the first step of conversion of ammonium into nitrite (${NH}_{4}^{+}\to {NO}_{2}^{-}$) and the nitrite-oxidizing bacteria (NOB), that perform the second step of oxidation of nitrites into nitrates (${NO}_{2}^{-}\to {NO}_{3}^{-}$; Xia et al., 2011). The nitrification inhibition test is widely used to monitor the effects of chemicals on soils (Pannu et al., 2012; Ruyters et al., 2013) because it is considered to be one of the most sensitive tests among the microbial exotoxicity assays (Reynolds et al., 1987; UBA, 1993; Ren and Frymier, 2003).

Even if widely applied in environmental toxicology, the nitrification test has never been applied for the evaluation of the effects of biodegradable plastics in soil under controlled laboratory conditions. In this context we are trying to increase the array of ecotoxicity tests applied in standard testing schemes in order to cover microorganisms besides plants and animals. For this purpose, we combine the test method for the evaluation of biodegradation of plastics in soil (ISO 17556, 2012) with the nitrification inhibition test (ISO 14238, 2012). First, biodegradation of mulch film is followed under controlled laboratory conditions and using high test material concentrations (>1% w/w); then, the resulting soil is tested for nitrification activity in order to verify possible effects of plastics biodegradation on soil health. The purpose of this approach is to determine the possible long-lasting effects of biodegradation of plastics on soil health. The assessment of the toxicity of plastics before biodegradation and the assessment of transient effects during biodegradation are out of the scope. For a discussion on the best conditions to perform eco-toxicity testing of biodegradable plastics see Degli-Innocenti (2014).

The applied test material loading in ISO 17556 (2012) is much higher than the expected application loading of biodegradable plastics in soil. For example: a typical biodegradable plastic film for mulching is 1.5 × 10^-5^ m thick and has a density of 1,250 kg m^-3^. This means 1.88 × 10^-2^ kg m^-2^ for one application. The soil depth where the plastic is typically used or remains after use is presumed to be 0.30 m, in agreement with the normal depth of soil tillage. Therefore, 1 m^2^ of plastic film covering 1 m^2^ of soil surface will typically be mixed with a volume of soil equal to 0.3 m^3^. This amount of soil weighs approximately 450 kg, considering a soil bulk density of 1500 kg m^-3^. Therefore, the typical loading of the plastic film in normal use will be approximately 0.0042% (1.88 × 10^-2^ kg/450 kg × 100).

## MATERIALS AND METHODS

### TEST MATERIAL

The test material is a black mulch film made with Mater-Bi DF04P, a biodegradable plastic material based on corn starch and biodegradable copolyesters (Novamont). Before biodegradation testing the mulch film was powdered by means of cryogenic grinding with liquid nitrogen using an IKA M20 grinder. The elemental analysis of this film was C = 61.00% N < 0.1% and H = 6.84% (analysis performed by Redox snc, Monza, Italy).

### REFERENCE MATERIAL

The reference material was micro-crystalline cellulose (Merck) in powder, as prescribed by the standard test method ISO 17556 (2012). The elemental analysis was C = 44.40%; N < 0.1; H = 6.00% (analysis performed by Redox snc, Monza, Italy).

### BIODEGRADATION TEST

The biodegradation test was carried out using the ISO 17556 (2012) test method. The test material is mixed with the selected soil. The mixture is allowed to stand in the test flask under controlled conditions over a period of time during which the carbon dioxide evolved is determined. In parallel, the background CO_2_ production is determined in a different flask containing soil without test material. The evolved carbon dioxide is determined at intervals by analyzing the carbon dioxide in air by an appropriate analytical method. The level of biodegradation expressed in per cent is determined by comparing the net carbon dioxide evolved with the theoretical amount (amount expected in case of total oxidation of the test material).

The soil was collected from an agricultural field at the Centro Sperimentazione ed Assistenza Agricola (CeRSAA) in Albenga (Italy). The soil is routinely analyzed by CeRSAA and has a C/N ratio of 9.2. The soil was screened with a 5 mm sieve.

The soil was then supplemented as described in **Table 1**, following the ISO 17556 (2012).

**Table 1:** Preparation of soil.

Constituent	Grams
Soil	1000
Mature compost screened <5 mm with a 50% water content	40
KH_2_PO_4_	0.2
MgSO_4_	0.1
NaNO_3_	0.4
Urea	0.2
NH_ 4_Cl	0.4
H_2_O	78 ml

The total solids were determined after drying in an oven at 105°C until constant mass was achieved. The volatile solids were determined after calcination at 550°C until constant mass was achieved. The pH was determined by diluting the soil in distilled water. A 10 g sample of soil was mixed with 25 ml of deionized water, stirred for 15 min and left unstirred for 30 min before measuring the pH with a Hanna Instruments pHmeter model pH 211 (Violante and Adamo, 2000).

According to ISO 17556 (2012), the pH of the inoculum should be between 6 and 8 and the C/N ratio should be at least 40:1. Both conditions were met.

### BIODEGRADATION TEST APPARATUS

The reactors are 3-L glass flasks. The temperature of the reactors is kept at 28°C ± 2°C. Pressurized air is sent over a gas flow controller and an air flow rate of about 6 L/h is supplied at the bottom of the reactors (dynamic conditions). The air flow and the CO_2_ concentration at each reactor outlet is automatically measured by means of an infra-red gas sensor (EC400 Eco-control Vimercate, Milan, Italy) and a mass flowmeter (Brooks Instruments, Model 5860S).

The CO_2_ evolution rate is calculated by multiplying the CO_2_ concentration (g/L) by the air flow rate (L/h). The amount of CO_2_ produced during two measurements is estimated by multiplying the CO_2_ evolution rate by the elapsed time from last measurement. The mineralization percentage is the ratio between the total net CO_2_ produced by the sample and the amount produced in the case of a complete transformation of its carbon into CO_2_.

### BIODEGRADATION TEST SET-UP

A set of seven reactors was used. The test set-up is shown in **Table 2**.

**Table 2:** Biodegradation test set-up.

Reactor No.	Test or reference material	Mass of test or reference material (g)	Mass of supplemented soil (g)
R 5	Blank	0	800.0
R 6	Blank	0	800.0
R 11	Mater Bi DF04P	10.0	800.0
R 12	Mater Bi DF04P	10.0	800.0
R 13	Mater Bi DF04P	10.0	800.0
R 14	Cellulose	10.0	800.0
R 15	Cellulose	10.0	800.0

### NITRIFICATION TEST

The nitrification test was carried out using the ISO 14238 (2012) test method. The rates or extent of N-mineralization in aerobic soils are determined by measuring the concentrations of ammonium, nitrite, and nitrate released during mineralization of an added nitrogenous organic compound. The influence of plastic biodegradation on N-mineralization is determined by amending soil with a readily degradable source of organic nitrogen, and measuring the percentage inhibition of product formation in soil where plastics have been biodegraded as compared to an untreated control.

The soil samples used in this test derived from the biodegradation test (from Reactors R6, R13, and R15; see **Table 2**). After the biodegradation phase, soils are carefully crumbled and supplemented with 100 mg/kg dry soil of (NH_4_)_2_SO_4_ in the form of powder. In practice 275.86 g of wet soil (water content 13%) were carefully mixed with 112.8 mg of (NH_4_)_2_SO_4_. Soil sample is stored in the dark at room temperature for 12 h to let the salt grains dissolve into the soil and to reach a homogeneous concentration. After this period, subsamples of 15 g (dry mass) are withdrawn and each placed in a 100 ml beaker; the beakers are then closed with Parafilm and incubated in the dark at 20°C ± 2°C until extraction. Once a week each sample is weighed in order to adjust water content to 13% and mixed to allow aeration of the soil.

At day 1, 8, 15, 22, and 29 after the addition of (NH_4_)_2_SO_4_, extraction is performed by mixing one soil subsample (15 g as dry mass) with 75 ml of a 1 M KCl solution in a 100 ml beaker. The slurry is stirred for 1 h with a magnetic stirrer and then centrifuged at 6000 rpm for 10 min. The supernatant is collected, stored at -20°C and sent for analyses.

The concentration of NH_4_, nitric-N, nitrous-N, was analyzed by CHEMSERVICE S.r.l. (Novate Milanese, Milan, Italy) by using the standard methods of the Italian Water Research Institute (IRSA) and APAT [Agenzia per la Protezione dell’Ambiente e per i Servizi Tecnici; Istituto di Ricerca sulle Istituto di Ricerca sulle Acque (IRSA), 2014a]. In particular: N-NH_4_ with the Method 4030 A2– Ammonia Nitrogen, Spectrophotometric determination by means of Nessler reagent [Istituto di Ricerca sulle Acque (IRSA), 2014b], nitric nitrogen with the Method 4020 – Determination of anions (fluoride, chloride, nitrite, bromide, nitrate, phosphate, and sulfate) by ion chromatography [Istituto di Ricerca sulle Acque (IRSA), 2014c], nitrous nitrogen with the Method 4050 – Nitrous nitrogen [Istituto di Ricerca sulle Acque (IRSA), 2014d]. The expanded uncertainty of measurement was ±15% and ±4% for N-NO_3_ and N-NH_4_, respectively.

### STATISTICAL ANALYSIS

The data were analyzed with Statgraphics Centurion XVI software (Statpoint Technologies, Inc. USA), by using the “comparison of regression lines” and the “Conditional Sums of Squares (ANOVA)” options.

## RESULTS

### BIODEGRADATION

The soil used in the biodegradation test had the following characteristics: total solids = 88.46% ± 0.17 (average of 3 determinations ± SD); volatile solids = 4.96% ± 0.06 (average of 3 determinations ± SD); pH = 7.48.

**FIGURE 1:**
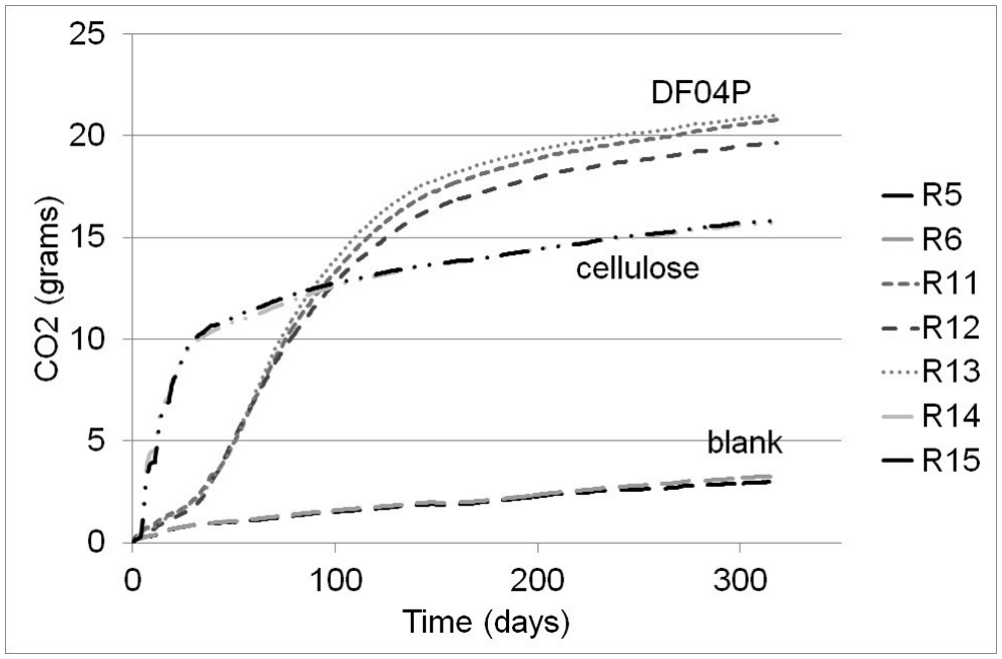
**CO_2_ cumulative evolution from the different reactors.** R5 and R6 = blank; R11, R12, and R13 Mater-Bi; R14 and R15 cellulose.

The total cumulative CO_2_ production for each reactor is shown in **Figure 1**. The different replicates showed a regular course and a very limited variance.

**FIGURE 2:**
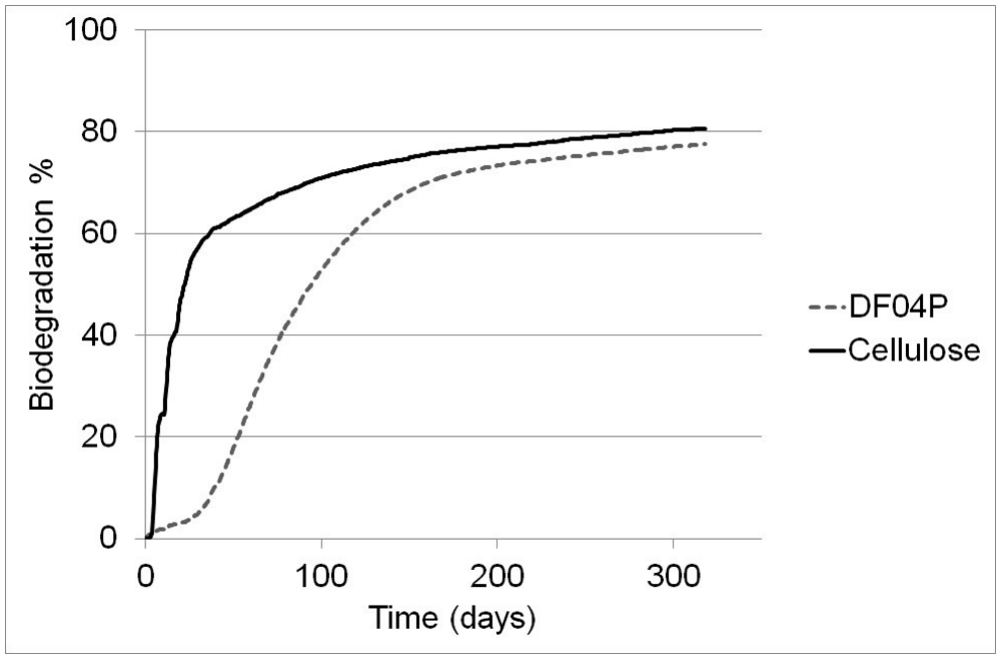
**Biodegradation of Mater-Bi DF04P (average of three replicates) and microcrystalline cellulose (average of two replicates)**.

The biodegradation percentage averages are plotted in **Figure 2**. Cellulose showed a very fast biodegradation phase that lasted about 50 days, followed by a progressive plateauing. Mater-Bi DF04P showed a lag phase of about 25 days followed by a steady biodegradation phase plateauing after 150 days. After 318 days, the biodegradation of both test material and reference material had reached a clear plateau. The single replicates of Mater-Bi had reached the following biodegradation percentages: R11 = 78.88%; R12 = 73.74%; R13 = 79.91%. The single replicates of cellulose were R14 = 80.16%; R15 = 80.92%.

According to ISO 17556 (2012), the test is considered valid when: (a) the degree of biodegradation of the reference material is more than 60% at the plateau phase or at the end of the test; and (b) the amount of carbon dioxide evolved from the three blanks is within 20% of the mean at the plateau phase or at the end of the test. Cellulose reached the 60% biodegradation threshold after 38 days. For technical reasons, only two blank replicates were used; at the end of the tests the two replicates were comparable: 15.73 and 15.85 g of CO_2_. We therefore consider the test as valid.

After 318 days the CO_2_ measurements were discontinued. Reactor R6 (blank), R13 (Mater-Bi DF04P), and R15 (cellulose) were maintained for further testing. The pH of soils was: reactors R6 (blank) = 7.96, R13 (Mater-Bi DF04P) = 8.29, and R15 (cellulose) = 8.15. The soil samples were then stored in the dark at room temperature for 7 months without any further CO_2_ measurement. Three times per week the content was routinely mixed and wet with deionized water to keep the original water content and maintain aerobic conditions until the nitrification experiment. Therefore, the biodegradation phase (both with and without CO_2_ monitoring) lasted 528 days in total.

The carbon balance of reactors R13 (Mater-Bi DF04P) and R15 (cellulose) is shown in **Table 3**. The residual carbon not mineralized into CO_2_ can be: (i) still bound in the original non-biodegraded material (C_plastic_), (ii) trapped in biomass (C_biomass_), (iii) converted into some by-products (C_residue_), as per Eq. (1).

**Table 3:** Carbon balance of reactors R13 and R15.

Reactor	Material	Amount g per reactor	Carbon content %	Carbon available for biodegradation g per reactor	Mineralization %	Carbon converted into CO_2_ g	Residual carbon g
R15	Cellulose	10.0	44.4	4.44	80.93	3.59	0.85
R13	Mater-Bi DF05P	10.0	61.0	6.10	79.91	4.87	1.22

### NITRIFICATION EXPERIMENT

**FIGURE 3:**
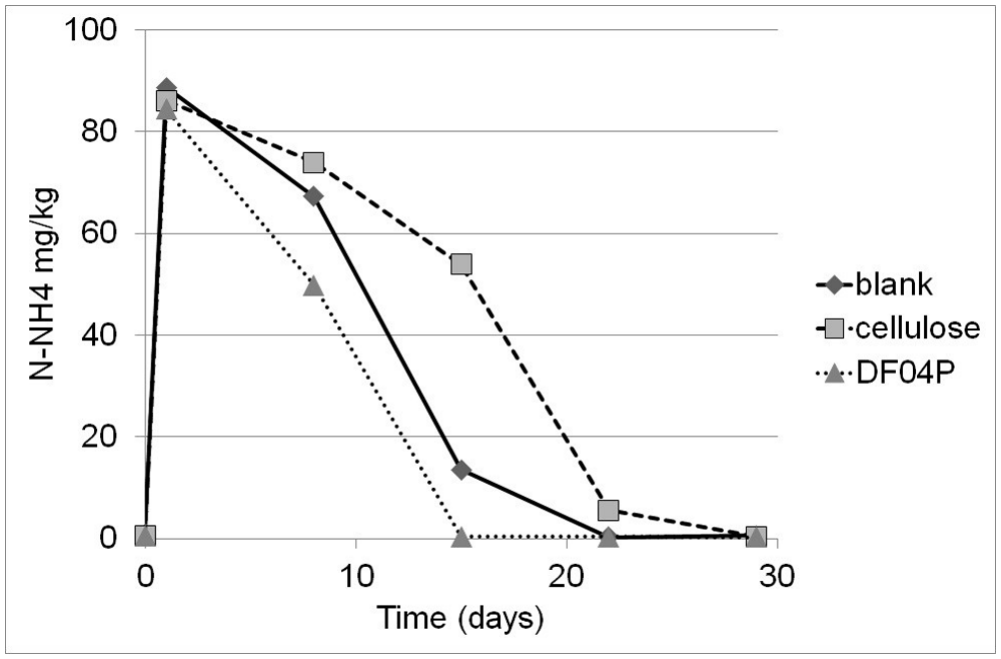
**The original concentration of N-NH_4_ immediately before test start was 1.0 (blank), 0.6 (cellulose), and 0.6 (Mater-Bi DF04P) mg/kg.** An amount of 100 mg/kg of N-NH_4_ was added at time zero and the NH_4_ depletion courses were followed once a week for 29 days. Each point is the average of three measurements; with the exception of time zero (two measurements). SE of the means is 3.4, maximum.

After the biodegradation phase, at the beginning of the nitrification test, the pH of blank, DF04F, and cellulose soils was 8.08, 8.18, and 8.03, respectively. The nitrification experiment was carried out as described in Materials and Methods and ran for 29 days. The N-NH_4_ concentrations are plotted in **Figure 3**.

The amount of nitrogen added as ammonium was 100 mg/kg of dry soil. The ammonium was fast consumed in all three soils, returning to the original level in 15–22 days. A linear regression analysis was performed from day 1 to 15 for the Mater-Bi DF04P and from day 1 to 22 for the blank and cellulose (**Table 4**). The ammonium depletion rates of the three soils differ significantly: Mater-Bi DF04P > blank soil > cellulose soil. The regression analysis and the applied statistical test indicated that there are statistically significant differences among the slopes for the various values of soil type at the 95% confidence level.

**Table 4:** Linear regression analysis of ammonium depletion courses.

Soil type	Intercept	Slope (mg N kg^-1^ d^-1^)	*R^2^*
Mater Bi DF04P	92.8	-6.0	0.97
Blank	94.8	-4.5	0.93
Cellulose	97.7	-3.7	0.85

**FIGURE 4:**
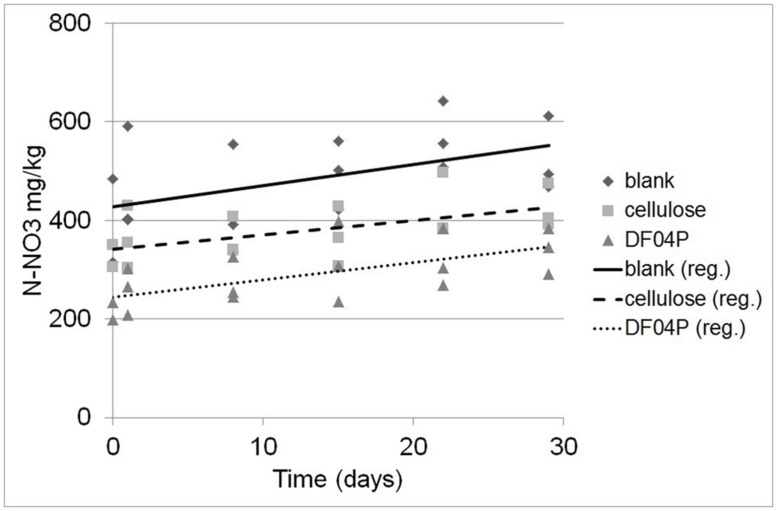
**Determination of N-NO_3_ concentration at different times after NH_4_ addition and the corresponding regression lines**.

The depletion of ammonium was accompanied by the appearance of nitrate. The nitrate measurements turned out to be quite variable (**Figure 4**) as a consequence of the higher expanded uncertainty of the N-NO_3_ measurement (see Materials and Methods). The result of a linear regression analysis is shown in **Table 5**.

**Table 5:** Linear regression analysis of NO_3_ formation courses.

Soil type	Intercept	Slope (mg N kg^-1^ d^-1^)	*R^2^*
DF04P	244.4	3.52	0.389
Blank	427.9	4.29	0.236
Cellulose	342.1	2.88	0.307

The regression analysis and the applied statistical tests (see Materials and Methods) indicate that there is a statistically significant relationship between the N-NO_3_ concentration and the time at the 95.0% confidence level. Differences among the slopes (N-NO_3_ formation rates) are not significant while differences among the intercepts are significant (at the 95% confidence level).

Considering the difference between the N-NO_3_ concentrations estimated at time 0 and time 29 (see **Table 6**), 102.2, 124.6, and 83.8 mg/kg of new N-NO_3_ was formed in Mater-Bi DF04P soil, blank soil, and cellulose soil, respectively.

**Table 6:** The predicted values for N-NO_3_ using the regression analysis are shown with the 95.0% confidence intervals.

Time (days)	Soil type	Predicted N-NO_3_ (mg/kg)	95.00% Confidence limits	
			Lower	Upper
0	DF04P	244.4	194.2	294.6
0	Blank	427.9	377.7	478.0
0	Cellulose	342.1	291.9	392.3
29	DF04P	346.6	290.3	402.9
29	Blank	552.5	496.2	608.8
29	Cellulose	425.9	369.6	482.2

No N-NO_2_ was detected in any sample with the exception of a spike of 8.5 mg/kg in the Mater-Bi DF04P sample at day 8.

## DISCUSSION

A mulch film made with Mater-Bi DF04P was tested for biodegradation using the standard test method ISO 17556 (2012). This test method requires a high initial dose of test material, that is, at least 100 times higher than the concentration applied to agricultural soil under the normal usage of mulch film. This makes ISO 17556 (2012) also suitable to prepare samples for ecotoxicity testing, where high concentrations of test material are desired in order to maximize the chance of detecting possible negative effects. Biodegradation of Mater-Bi DF04P and cellulose (used as the positive reference) reached a plateau phase after about 200 days (**Figure 2**) and biodegradation rates were negligible at day 318, when it was decided to discontinue the CO_2_ measurements, because a test elongation would not have added more information. Three reactors, one for each experimental group (cellulose, DF04P, and blank) were not discarded but maintained under constant environmental conditions (humidity and aeration) for a further 7 months without any CO_2_ measurement, until the nitrification test. Therefore, the biodegradation phase of the samples subjected to the nitrification test lasted 528 days in total, within the maximum time prescribed by the standards for soil biodegradability (2 years maximum).

The mulch film showed high levels of mineralization in soil. The biodegradation relative to cellulose was 96.24% after 318 days. This is considered as proof of soil biodegradability by the Vinçotte “OK Biodegradable Soil” testing scheme and also by French standard NF U 52-001 (2005) and by Italian standards UNI 11462 (2012) and UNI 11495 (2013).

After the biodegradation phase, the different soils were tested for the nitrification ability considered to be a relevant parameter for monitoring soil quality. The nitrification test procedure derived from the International Standard ISO 14238 (2012). The results showed that the soil where the mulch film had been biodegraded did not inhibit nitrification in comparison with blank soil and soil where cellulose (a GRAS, Generally Recognized As Safe, substance) had degraded in parallel.

The ammonium depletion was total in all soils and the depletion rate in soil that had been supplemented with the biodegradable mulch film was even higher. The ammonium depletion can be caused by nitrification, microbial uptake, and loss. The ammonium depletion was accompanied by the appearance of newly formed NO_3_. A quite high dispersion of the NO_3_ values was found (**Figure 4**). This data scattering is due to the rather high expanded uncertainty of the N-NO_3_ measurement. This variability seems to be common in this kind of study (Ruyters et al., 2013) and must be addressed with a statistical approach. The experimental set up suggested by the International standard ISO 14238 (2012) was designed to prevent the establishment of anoxic conditions that would lead to denitrification. We do not have data to exclude that the observed variation of N-NO_3_ measurements was due to denitrification caused by the establishment of anoxic micro-environments within the soil samples.

NO_2_ was only detected in very small amounts in the Mater-Bi soil as a spike after 8 days of incubation. At the same time the oxidation of ammonium was at its culmination as shown in **Figure 3**. We consider this to be an indication that the first step (${NH}_{4}^{+}\to {NO}_{2}^{-}$) carried out by AOB is temporarily slightly faster than the second step (${NO}_{2}^{-}\to {NO}_{3}^{-}$) performed by nitrate-oxidizing bacteria (NOB).

The amount of newly formed N-NO_3_ is fairly consistent with the amount of N-NH_4_ added to the soils, suggesting that the added N-NH_4_ was indeed quantitatively converted into N-NO_3_ by means of nitrification.

No statistically significant differences were noticed in the NO_3_ formation rate among the three soils. This suggests that the soil nitrification activity was not affected by the biodegradation of the plastic mulch film.

On the other hand, the intercepts (i.e., the original NO_3_ concentration of the soils at time 0) are different. The NO_3_ concentration at the beginning of the nitrification experiment was higher in the blank soil and lower in the cellulose soil and even more so in the DF04P soil (see **Figure 4**; **Table 6**). This result at first glance is unexpected because the three soils derived from the same source, i.e., a well homogenized soil sample, distributed into the different reactors at the beginning of the biodegradation phase. Thus an identical NO_3_ concentration was also expected after 528 days. In order to explain these differences we make the hypothesis that NO_3_ was assimilated by microorganisms during the biodegradation phase and trapped in biomass. The addition of a biodegradable carbon source in a soil elicits microbial growth with formation of new biomass. As a consequence, also uptake and assimilation of NO_3_ are stimulated in order to cope with the biosynthesis of nitrogen compounds (e.g., amino acids, nucleosides). We can estimate the amount of NO_3_ needed for the biosynthesis of new biomass with some calculations based on the following assumptions. The first assumption is that all the residual carbon (**Table 3**) not mineralized into CO_2_ has been assimilated and used for the microbial anabolism. The second assumption is that 1 g of nitrogen is needed for 10 g of carbon assimilated into biomass, considering that the typical carbon/nitrogen ratio of microbial biomass is 10:1 (Pirt, 1975). The carbon of cellulose assimilated in biomass was 0.85 g (see **Table 3**). Applying the 1:10 ratio, 0.085 mg of nitrogen can be expected to be taken up from soil and assimilated together with this carbon. This amount corresponds to a concentration of 105.8 mg/kg of N-NO_3_ (85 mg in 800 g of soil; see **Table 2**). Subtracting this amount from the N-NO_3_ concentration of blank soil (428 mg/kg), an amount of 322 mg/kg is left, very similar to the concentration actually measured in cellulose soil after the biodegradation phase (342 mg/kg). Similarly, a theoretical concentration of 274 mg/kg of N-NO_3_, consistent with the experimental measurement (244 mg/kg), is found for Mater-Bi DF04P. There is good consistency between the N-NO_3_ concentration estimated by applying the hypothetical assumptions and the experimental values. This supports the hypothesis that the NO_3_ deficiency found in soils supplemented with a biodegradable carbon source in comparison with the blank soil is indeed the consequence of nitrogen assimilation caused by microbial growth and biomass formation. This indirectly also supports the idea that the residual, non-mineralized carbon is indeed converted into biomass.

To our knowledge, the present study is the first attempt to set up a laboratory approach based on the combination of two standard test methods: the biodegradation test (ISO 17556, 2012) for the measurement of biodegradation and the preparation of soil samples under controlled conditions and the ISO 14238 (2012) for the measurement of nitrification activity. The results of this work suggest that the addition of biodegradable plastics does not exert an inhibitory effect on the nitrification activity of soil even when the applied dose is 100 times higher than the normal concentration. The nitrifying capability of soil after biodegradation of such a high amount of biodegradable mass is not impaired. The absence of negative effects of Mater-Bi mulch film on soil nitrification capability is not unexpected considering that Mater-Bi biodegradable plastics: (i) are not toxic (for example the Acute Oral Toxicity of Mater-Bi in rats is DL_50_ > 2000 mg/kg (OECD 420, 2001), (ii) are composed of biodegradable monomers (Siotto et al., 2011, 2012), and (iii) tend to be either in a solid inert form (before degradation) or, after enzymatic depolymerization, tend to be rapidly mineralized (Saponaro et al., 2008).

Effects on the nitrification activity of soils mulched with bioplastics were reported by Chenon et al. (2012). However, the experiments were carried out on soil samples taken from not fully characterized fields where biodegradable mulches had been applied. Field testing requires an extended experimental design and a methodological and statistical approach similar to that applied in agronomical studies in order to cope with the many environmental factors that can undermine the establishment of a credible cause-effect relationship. The laboratory approach described in this Technology Report drastically reduces the uncertainty by imposing strictly controlled conditions.

## CONCLUSION

The aim of this study was to monitor mulch film biodegradation, to prepare soil samples for nitrification tests under laboratory controlled conditions and to apply the nitrification test after substantial biodegradation of the test material and in any case within the 2-year testing period prescribed by the standards.

We consider the test approach applied in this work to be suitable for reaching conclusions on the effects of biodegradable plastics on soil for the following reasons:

(1) A unique, well homogenized soil is either mixed with the test material or used as blank; the soils are then treated in parallel, the only difference being the test material. The effects, if any, cannot be reasonably ascribed to poor sampling design or other uncontrolled factors.
(2) The test materials are well characterized and added by the practitioner under controlled conditions.
(3) Doses are set by the practitioner. Very high concentrations can be tested in order to be able to detect even very mild effects.
(4) Test conditions are well controlled.
(5) Substances released during biodegradation cannot be lost by leaching because the system is closed and hence any solid or liquid substance formed during biodegradation is either degraded or it builds up in the test system. If any substance that could affect soil activity were produced, this would be detected on the basis of test sensitivity.

The test approach applied in this work seems suitable for the application in the sector of biodegradable plastics but it needs to be replicated with other plastic materials and other soils in order to be validated.

## Conflict of Interest Statement

The authors declare that the research was conducted in the absence of any commercial or financial relationships that could be construed as a potential conflict of interest.
